# Supplementary material for: Longitudinal trajectories of blood lipid levels in an ageing population sample of Russian Western-Siberian urban population
Source: PLoS One. 2021 Dec 2;16(12):e0260229. doi: 10.1371/journal.pone.0260229 (PMC8638938; doi:10.1371/journal.pone.0260229)
Supplement: S1 Table — Baseline total cholesterol, LDL-C, HDL-C and triglycerides (intercept) and change in LDL-C, HDL-C and triglycerides per year (slope) in the 5-yr cohorts (unadjusted). (DOCX) [file pone.0260229.s001.docx]

**Table S1.** Complete cases of all three waves (N = 2009). Baseline total cholesterol, LDL-C, HDL-C and triglycerides (intercept) and change in LDL-C, HDL-C and triglycerides per year (slope) in the 5-yr cohorts (unadjusted).

|  |  | Age range in W1 | **TC** | | | **LDL-C** | | | **HDL-C** | | | **TG** | | |
| --- | --- | --- | --- | --- | --- | --- | --- | --- | --- | --- | --- | --- | --- | --- |
|  |  |  | coeff. | SE | p-value | coeff. | SE | p-value | coeff. | SE | p-value | coeff. | SE | p-value |
| Intercept | Estimate (mmol/l) | 45-49 (ref) | 5.91 | 0.059 | <0.001 | 3.76 | 0.052 | <0.001 | 1.53 | 0.016 | <0.001 | 1.36 | 0.039 | <0.001 |
|  | Difference compared to reference group | 50-54 | 0.368 | 0.080 | <0.001 | 0.280 | 0.071 | <0.001 | 0.018 | 0.022 | 0.415 | 0.154 | 0.053 | 0.004 |
|  |  | 55-59 | 0.365 | 0.077 | <0.001 | 0.284 | 0.068 | <0.001 | 0.008 | 0.021 | 0.691 | 0.158 | 0.051 | 0.002 |
|  |  | 60-64 | 0.555 | 0.084 | <0.001 | 0.506 | 0.075 | <0.001 | -0.013 | 0.023 | 0.580 | 0.138 | 0.055 | 0.013 |
|  |  | 65-69 | 0.380 | 0.087 | <0.001 | 0.341 | 0.077 | <0.001 | 0.009 | 0.024 | 0.724 | 0.066 | 0.057 | 0.246 |
| Slope | Estimate (mmol/l/year) | 45-49 (ref) | -0.021 | 0.005 | <0.001 | -0.011 | 0.005 | 0.015 | -0.016 | 0.001 | <0.001 | 0.015 | 0.003 | <0.001 |
|  | Difference compared to reference group | 50-54 | -0.035 | 0.007 | <0.001 | -0.026 | 0.006 | <0.001 | -0.002 | 0.002 | 0.176 | -0.011 | 0.004 | 0.006 |
|  |  | 55-59 | -0.049 | 0.007 | 0.001 | -0.042 | 0.006 | <0.001 | 0.003 | 0.002 | 0.874 | -0.017 | 0.004 | <0.001 |
|  |  | 60-64 | -0.067 | 0.007 | <0.001 | -0.056 | 0.007 | <0.001 | 0.002 | 0.002 | 0.356 | -0.028 | 0.005 | <0.001 |
|  |  | 65-69 | -0.068 | 0.008 | <0.001 | -0.055 | 0.007 | <0.001 | -0.002 | 0.002 | 0.400 | -0.025 | 0.005 | <0.001 |
