# Supplementary material for: Longitudinal trajectories of blood lipid levels in an ageing population sample of Russian Western-Siberian urban population
Source: PLoS One. 2021 Dec 2;16(12):e0260229. doi: 10.1371/journal.pone.0260229 (PMC8638938; doi:10.1371/journal.pone.0260229)
Supplement: S2 Table — Baseline total cholesterol, LDL-C, HDL-C and triglycerides (intercept) and change in LDL-C, HDL-C and triglycerides per year (slope) in the 5-yr cohorts. Adjusted by sex, lipid-lowering treatment, smoking, BMI, alcohol, education, marital status, CVD mortality, all causes mortality. (DOCX) [file pone.0260229.s002.docx]

**Table S2.** Complete cases of all three waves (N = 2009). Baseline total cholesterol, LDL-C, HDL-C and triglycerides (intercept) and change in LDL-C, HDL-C and triglycerides per year (slope) in the 5-yr cohorts.

|  |  | Age range in W1 | **TC** | | | **LDL-C** | | | **HDL-C** | | | **TG** | | |
| --- | --- | --- | --- | --- | --- | --- | --- | --- | --- | --- | --- | --- | --- | --- |
|  |  |  | coeff. | SE | p-value | coeff. | SE | p-value | coeff. | SE | p-value | coeff. | SE | p-value |
| Intercept | Estimate (mmol/l) | 45-49 (ref) | 5.28 | 0.103 | <0.001 | 3.30 | 0.093 | <0.001 | 1.49 | 0.031 | <0.001 | 1.07 | 0.072 | <0.001 |
|  | Difference compared to reference group | 50-54 | 0.351 | 0.078 | <0.001 | 0.263 | 0.070 | <0.001 | 0.039 | 0.021 | 0.065 | 0.101 | 0.050 | 0.044 |
|  |  | 55-59 | 0.340 | 0.074 | <0.001 | 0.261 | 0.067 | <0.001 | 0.029 | 0.021 | 0.157 | 0.105 | 0.048 | 0.029 |
|  |  | 60-64 | 0.537 | 0.083 | <0.001 | 0.484 | 0.074 | <0.001 | 0.012 | 0.023 | 0.605 | 0.087 | 0.054 | 0.106 |
|  |  | 65-69 | 0.381 | 0.086 | <0.001 | 0.332 | 0.077 | <0.001 | 0.037 | 0.024 | 0.118 | 0.019 | 0.056 | 0.727 |
| Slope | Estimate (mmol/l/year) | 45-49 (ref) | -0.020 | 0.005 | <0.001 | -0.010 | 0.005 | 0.040 | -0.015 | 0.001 | <0.001 | 0.011 | 0.003 | 0.001 |
|  | Difference compared to reference group | 50-54 | -0.032 | 0.007 | <0.001 | -0.024 | 0.006 | <0.001 | -0.003 | 0.002 | 0.104 | -0.011 | 0.004 | 0.012 |
|  |  | 55-59 | -0.046 | 0.007 | <0.001 | -0.039 | 0.006 | <0.001 | -0.001 | 0.002 | 0.522 | -0.013 | 0.004 | 0.001 |
|  |  | 60-64 | -0.065 | 0.007 | <0.001 | -0.054 | 0.007 | <0.001 | -0.000 | 0.002 | 0.898 | -0.020 | 0.005 | <0.001 |
|  |  | 65-69 | -0.067 | 0.007 | <0.001 | -0.054 | 0.007 | <0.001 | -0.003 | 0.002 | 0.043 | -0.013 | 0.006 | 0.024 |

Adjusted by sex, lipid-lowering treatment, smoking, BMI, alcohol, education, marital status, CVD mortality, all causes mortality
