# Supplementary material for: Longitudinal trajectories of blood lipid levels in an ageing population sample of Russian Western-Siberian urban population
Source: PLoS One. 2021 Dec 2;16(12):e0260229. doi: 10.1371/journal.pone.0260229 (PMC8638938; doi:10.1371/journal.pone.0260229)
Supplement: S3 Table — Baseline total cholesterol, LDL-C, HDL-C and triglycerides (intercept) and change in LDL-C, HDL-C and triglycerides per year (slope) in the 5-yr cohorts (unadjusted). (DOCX) [file pone.0260229.s003.docx]

**Table S3.** Selected sample after exclusion of subjects dropped out after baseline examination and did not die during follow-up (N = 7,606). Baseline total cholesterol, LDL-C, HDL-C and triglycerides (intercept) and change in LDL-C, HDL-C and triglycerides per year (slope) in the 5-yr cohorts (unadjusted).

|  |  | Age range in W1 | **TC** | | | **LDL-C** | | | **HDL-C** | | | **TG** | | |
| --- | --- | --- | --- | --- | --- | --- | --- | --- | --- | --- | --- | --- | --- | --- |
|  |  |  | coeff. | SE | p-value | coeff. | SE | p-value | coeff. | SE | p-value | coeff. | SE | p-value |
| Intercept | Estimate (mmol/l) | 45-49 (ref) | 5.90 | 0.035 | <0.001 | 3.73 | 0.031 | <0.001 | 1.54 | 0.010 | <0.001 | 1.39 | 0.022 | <0.001 |
|  | Difference compared to reference group | 50-54 | 0.241 | 0.047 | <0.001 | 0.200 | 0.042 | <0.001 | -0.002 | 0.022 | 0.880 | 0.097 | 0.030 | 0.001 |
|  |  | 55-59 | 0.348 | 0.046 | <0.001 | 0.286 | 0.041 | <0.001 | -0.009 | 0.021 | 0.494 | 0.155 | 0.029 | <0.001 |
|  |  | 60-64 | 0.509 | 0.047 | <0.001 | 0.455 | 0.042 | <0.001 | -0.018 | 0.023 | 0.180 | 0.161 | 0.030 | <0.001 |
|  |  | 65-69 | 0.429 | 0.045 | <0.001 | 0.389 | 0.040 | <0.001 | -0.028 | 0.024 | 0.031 | 0.154 | 0.029 | <0.001 |
| Slope | Estimate (mmol/l/year) | 45-49 (ref) | -0.025 | 0.004 | <0.001 | -0.013 | 0.003 | 0.015 | -0.017 | 0.001 | <0.001 | 0.012 | 0.002 | <0.001 |
|  | Difference compared to reference group | 50-54 | -0.027 | 0.005 | <0.001 | -0.022 | 0.004 | <0.001 | -0.002 | 0.001 | 0.166 | -0.006 | 0.003 | 0.047 |
|  |  | 55-59 | -0.047 | 0.005 | 0.001 | -0.041 | 0.004 | <0.001 | 0.000 | 0.001 | 0.800 | -0.015 | 0.003 | <0.001 |
|  |  | 60-64 | -0.066 | 0.005 | <0.001 | -0.054 | 0.005 | <0.001 | 0.001 | 0.001 | 0.355 | -0.028 | 0.003 | <0.001 |
|  |  | 65-69 | -0.070 | 0.005 | <0.001 | -0.059 | 0.005 | <0.001 | 0.002 | 0.001 | 0.108 | -0.030 | 0.003 | <0.001 |
