# Supplementary material for: Longitudinal trajectories of blood lipid levels in an ageing population sample of Russian Western-Siberian urban population
Source: PLoS One. 2021 Dec 2;16(12):e0260229. doi: 10.1371/journal.pone.0260229 (PMC8638938; doi:10.1371/journal.pone.0260229)
Supplement: S4 Table — Baseline total cholesterol, LDL-C, HDL-C and triglycerides (intercept) and change in LDL-C, HDL-C and triglycerides per year (slope) in the 5-yr cohorts. Adjusted by sex, lipid-lowering treatment, smoking, BMI, alcohol, education, marital status, CVD mortality, all causes mortalit. (DOCX) [file pone.0260229.s004.docx]

**Table S4.** Selected sample after exclusion of dropped out after baseline examination and did not die during follow-up (N = 7,606). Baseline total cholesterol, LDL-C, HDL-C and triglycerides (intercept) and change in LDL-C, HDL-C and triglycerides per year (slope) in the 5-yr cohorts.

|  |  | Age range in W1 | **TC** | | | **LDL-C** | | | **HDL-C** | | | **TG** | | |
| --- | --- | --- | --- | --- | --- | --- | --- | --- | --- | --- | --- | --- | --- | --- |
|  |  |  | coeff. | SE | p-value | coeff. | SE | p-value | coeff. | SE | p-value | coeff. | SE | p-value |
| Intercept | Estimate (mmol/l) | 45-49 (ref) | 5.31 | 0.067 | <0.001 | 3.33 | 0.059 | <0.001 | 1.47 | 0.019 | <0.001 | 1.10 | 0.045 | <0.001 |
|  | Difference compared to reference group | 50-54 | 0.262 | 0.048 | <0.001 | 0.209 | 0.043 | <0.001 | 0.024 | 0.013 | 0.077 | 0.063 | 0.031 | 0.041 |
|  |  | 55-59 | 0.357 | 0.047 | <0.001 | 0.286 | 0.042 | <0.001 | 0.024 | 0.013 | 0.068 | 0.101 | 0.030 | 0.001 |
|  |  | 60-64 | 0.531 | 0.051 | <0.001 | 0.456 | 0.045 | <0.001 | 0.011 | 0.014 | 0.429 | 0.139 | 0.032 | <0.001 |
|  |  | 65-69 | 0.486 | 0.051 | <0.001 | 0.433 | 0.045 | <0.001 | 0.017 | 0.014 | 0.219 | 0.078 | 0.032 | 0.016 |
| Slope | Estimate (mmol/l/year) | 45-49 (ref) | -0.025 | 0.004 | <0.001 | -0.013 | 0.003 | 0.040 | -0.015 | 0.001 | <0.001 | 0.008 | 0.002 | 0.001 |
|  | Difference compared to reference group | 50-54 | -0.026 | 0.005 | <0.001 | -0.022 | 0.004 | <0.001 | -0.003 | 0.001 | 0.042 | -0.005 | 0.003 | 0.106 |
|  |  | 55-59 | -0.045 | 0.005 | <0.001 | -0.039 | 0.004 | <0.001 | -0.001 | 0.001 | 0.305 | -0.011 | 0.003 | <0.001 |
|  |  | 60-64 | -0.064 | 0.005 | <0.001 | -0.052 | 0.005 | <0.001 | -0.001 | 0.001 | 0.487 | -0.024 | 0.003 | <0.001 |
|  |  | 65-69 | -0.068 | 0.005 | <0.001 | -0.058 | 0.005 | <0.001 | -0.000 | 0.001 | 0.870 | -0.022 | 0.003 | <0.001 |

Adjusted by sex, lipid-lowering treatment, smoking, BMI, alcohol, education, marital status, CVD mortality, all causes mortalit
