# Supplementary figures and images for: Longitudinal trajectories of blood lipid levels in an ageing population sample of Russian Western-Siberian urban population
Source: PLoS One. 2021 Dec 2;16(12):e0260229. doi: 10.1371/journal.pone.0260229 (PMC8638938; doi:10.1371/journal.pone.0260229)

**Figure S1.**

TC trajectories in men and women over the 12 years of follow up.


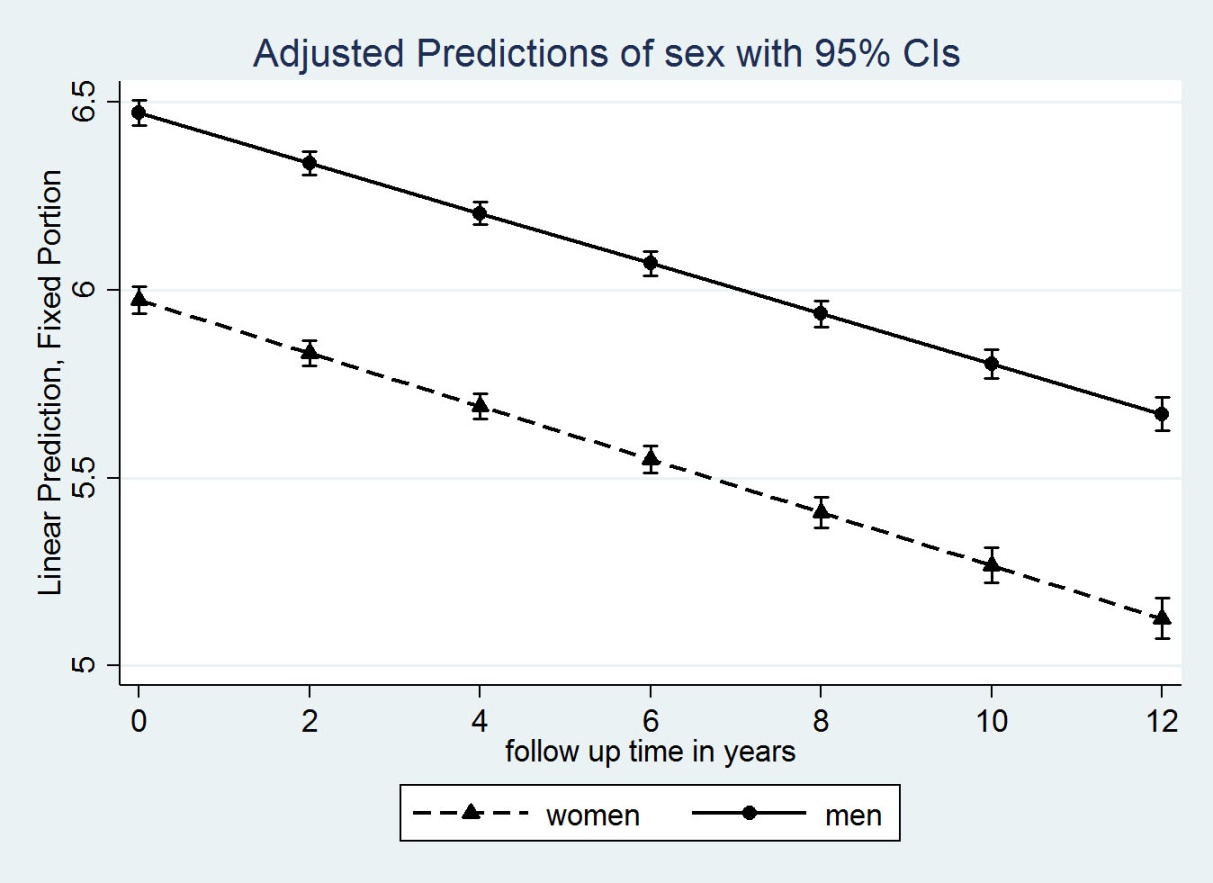

Supplement: S1 Fig — (DOCX) [file pone.0260229.s005.docx]

**Figure S2.**

LDL-C trajectories in men and women over the 12 years of follow up.


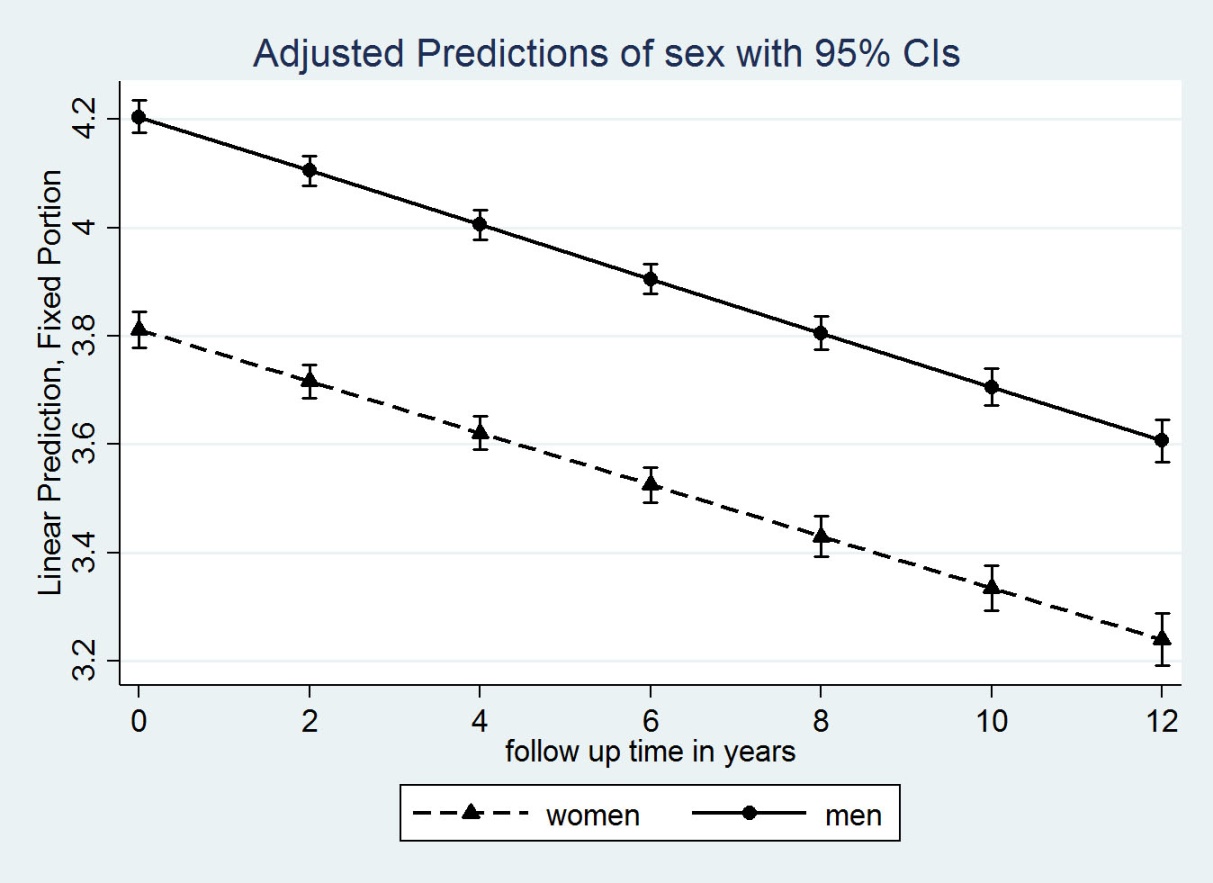

Supplement: S2 Fig — (DOCX) [file pone.0260229.s006.docx]

**Figure S3.**

HDL-C trajectories in men and women over the 12 years of follow up.


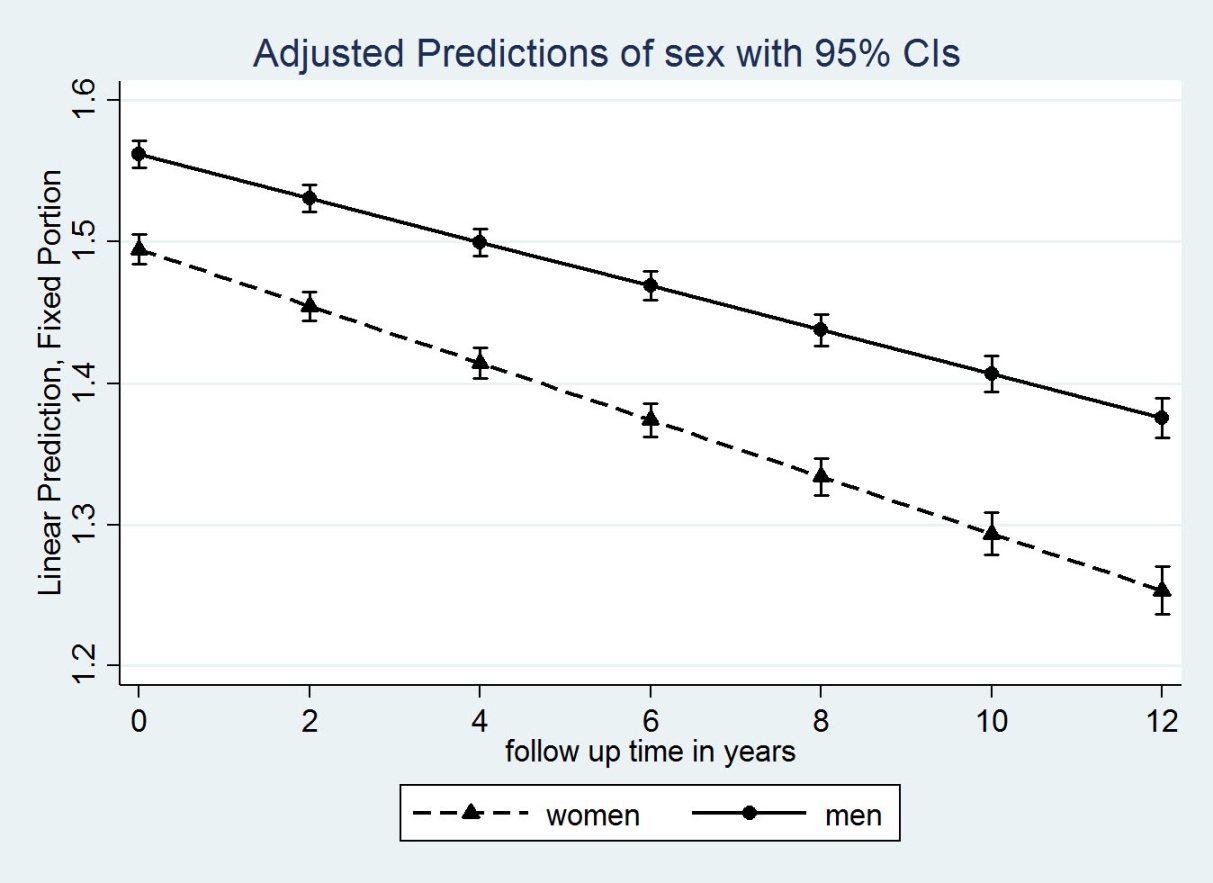

Supplement: S3 Fig — (DOCX) [file pone.0260229.s007.docx]

**Figure S4.**

TG trajectories in men and women over the 12 years of follow up.


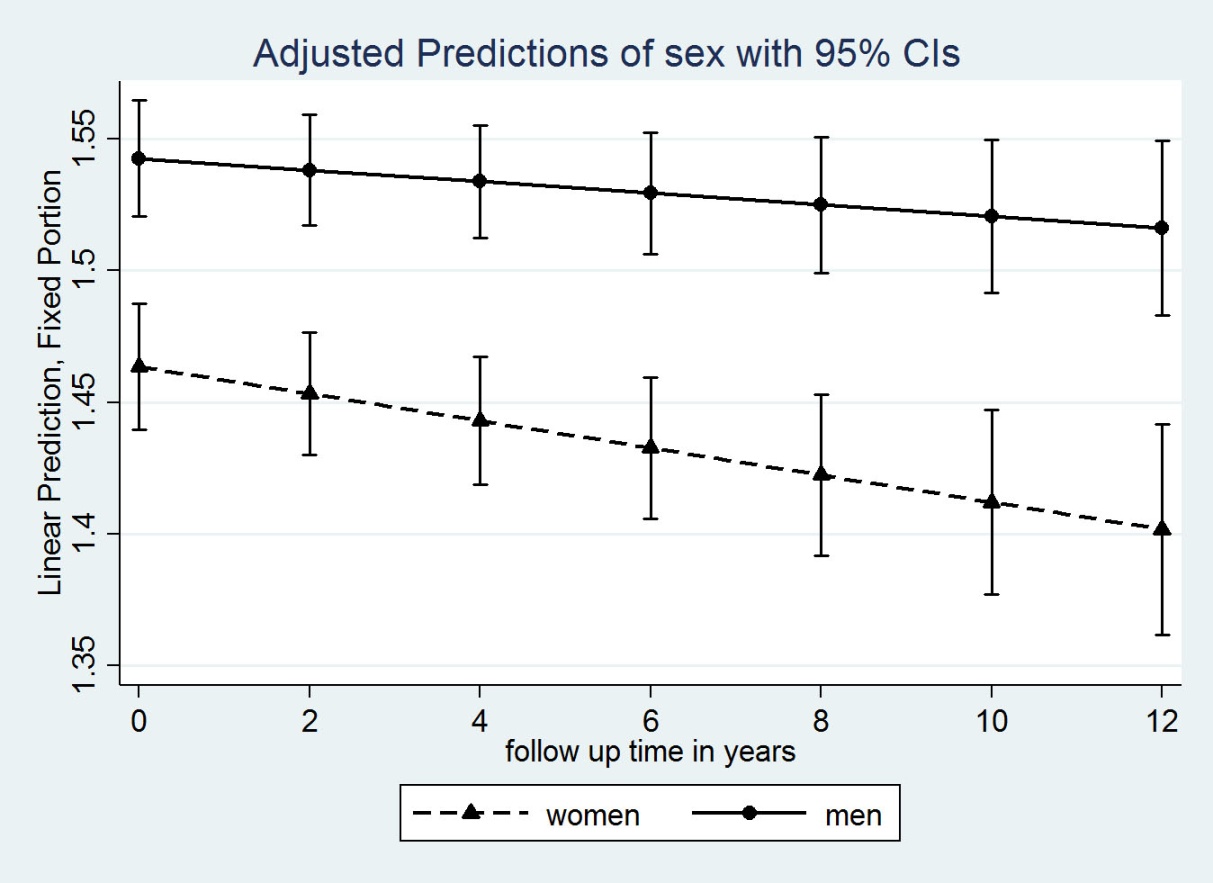

Supplement: S4 Fig — (DOCX) [file pone.0260229.s008.docx]

**Figure S5.**

Non HDL-C trajectories in men and women over the 12 years of follow up.


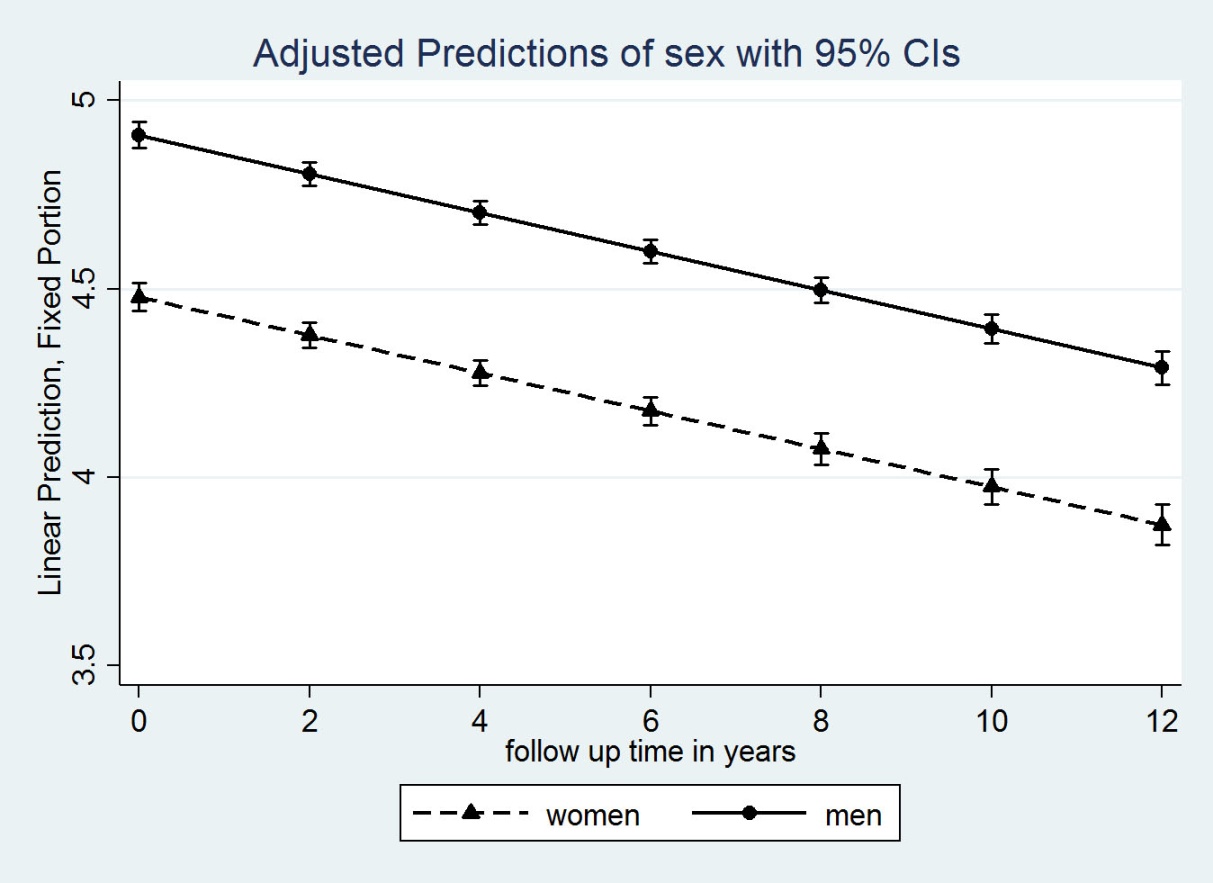

Supplement: S5 Fig — (DOCX) [file pone.0260229.s009.docx]

**Figure S6.**

Ratio LDL/HDL trajectories in men and women over the 12 years of follow up.


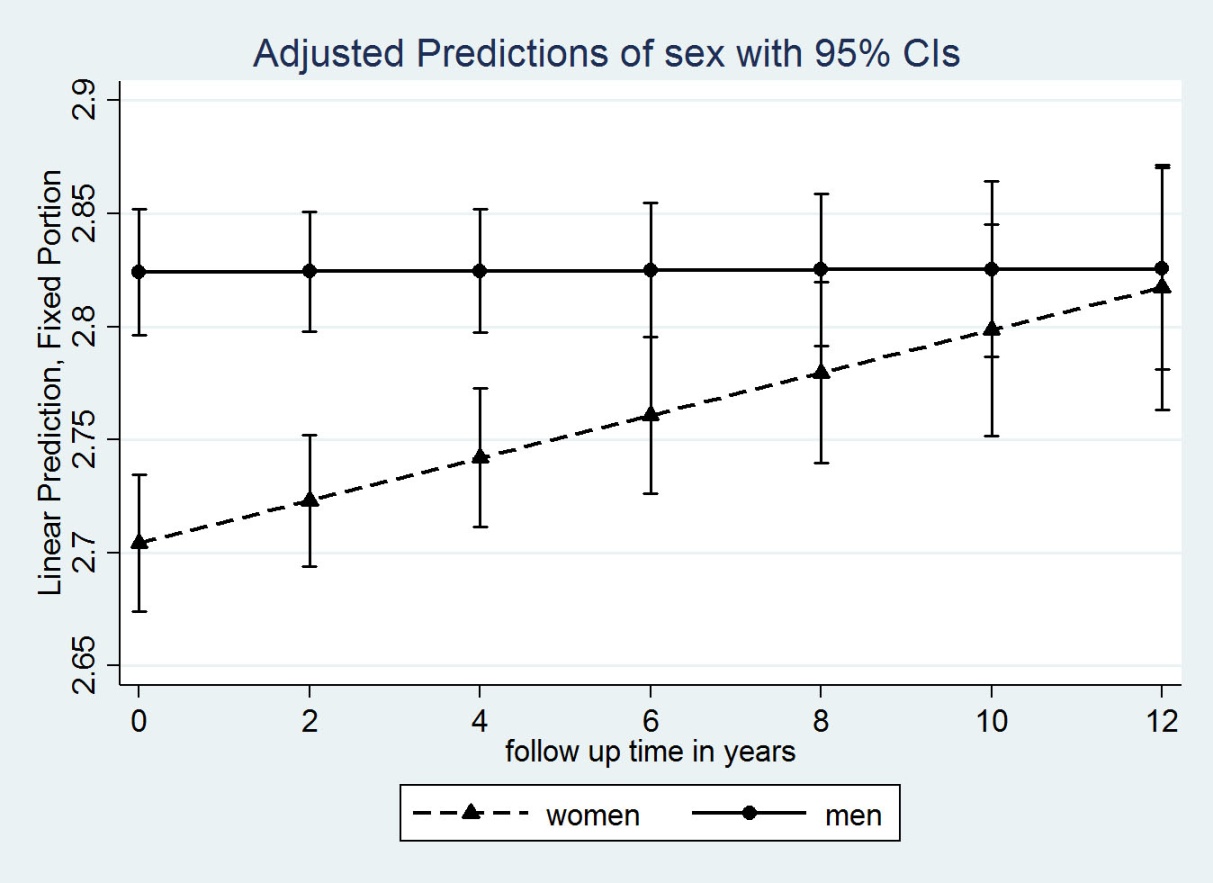

Supplement: S6 Fig — (DOCX) [file pone.0260229.s010.docx]

**Figure S7.**

TC trajectories in men and women over the 12 years of follow up by smoking.


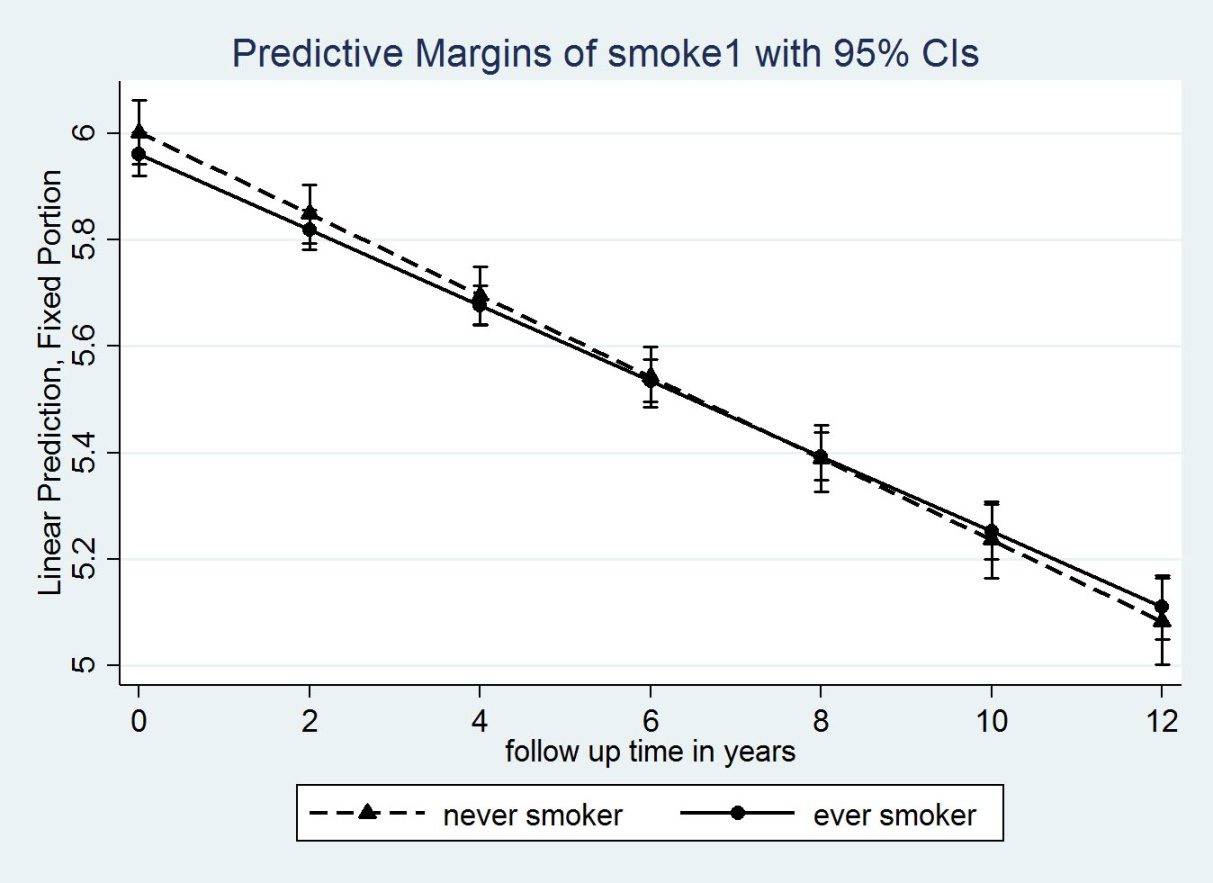

Supplement: S7 Fig — (DOCX) [file pone.0260229.s011.docx]

**Figure S8.**

LDL-C trajectories in men and women over the 12 years of follow up by smoking.


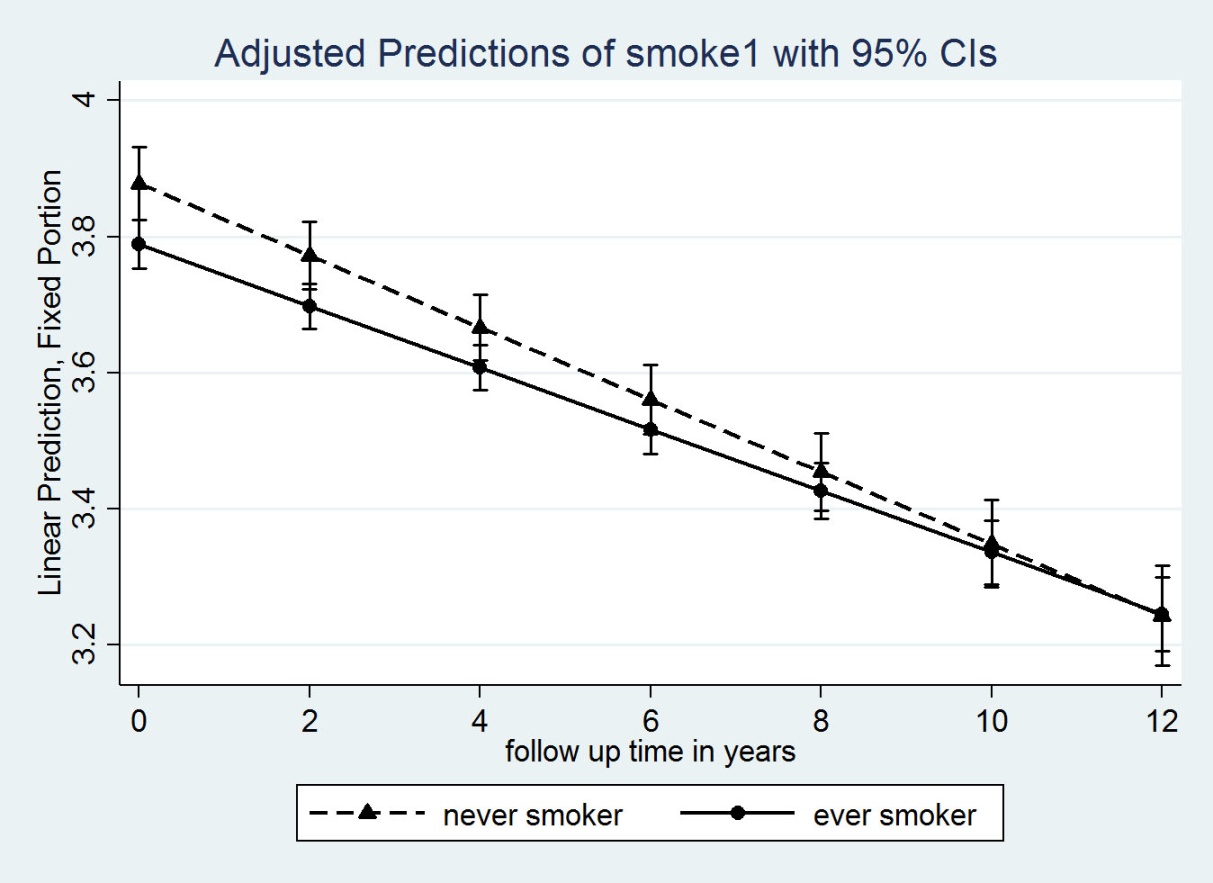

Supplement: S8 Fig — (DOCX) [file pone.0260229.s012.docx]

**Figure S9.**

HDL-C trajectories in men and women over the 12 years of follow up by smoking.


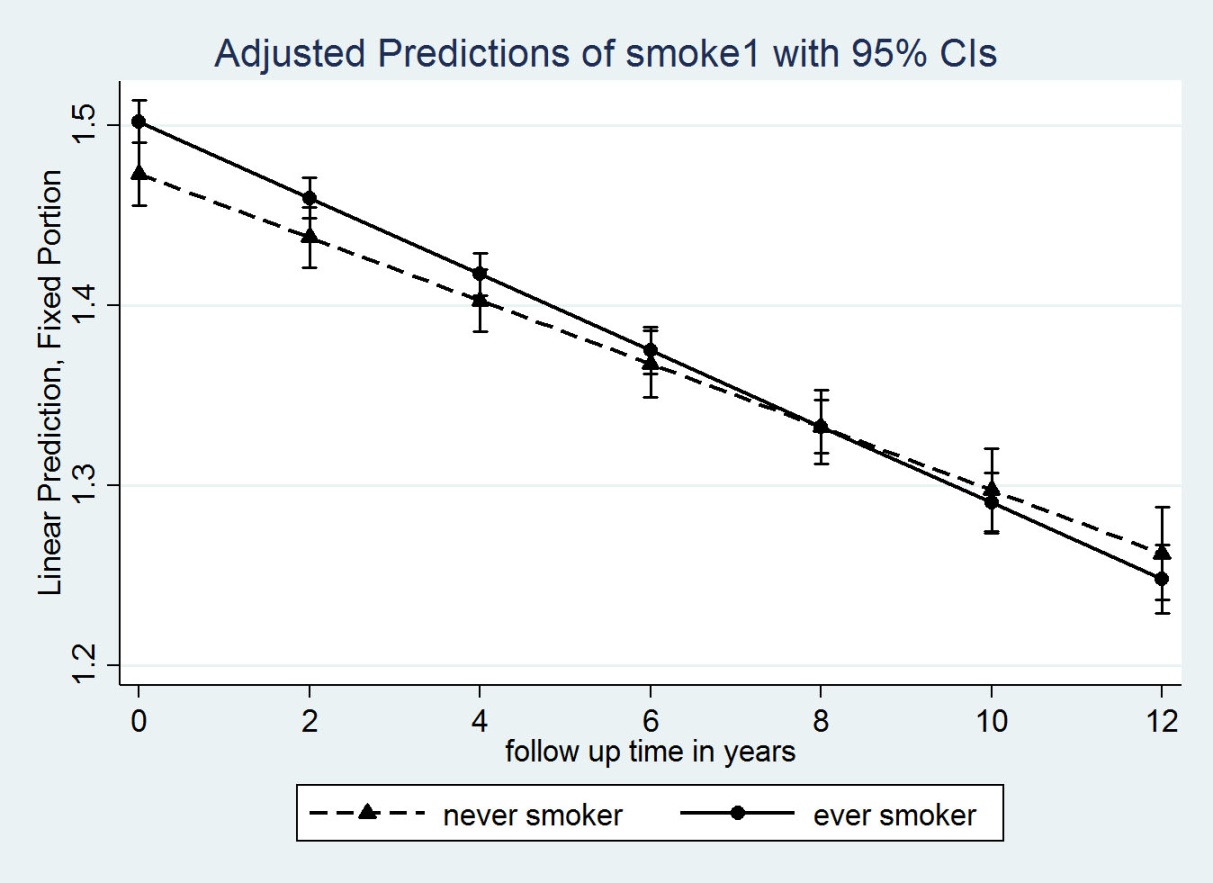

Supplement: S9 Fig — (DOCX) [file pone.0260229.s013.docx]

**Figure S10.**

TG trajectories in men and women over the 12 years of follow up by smoking.


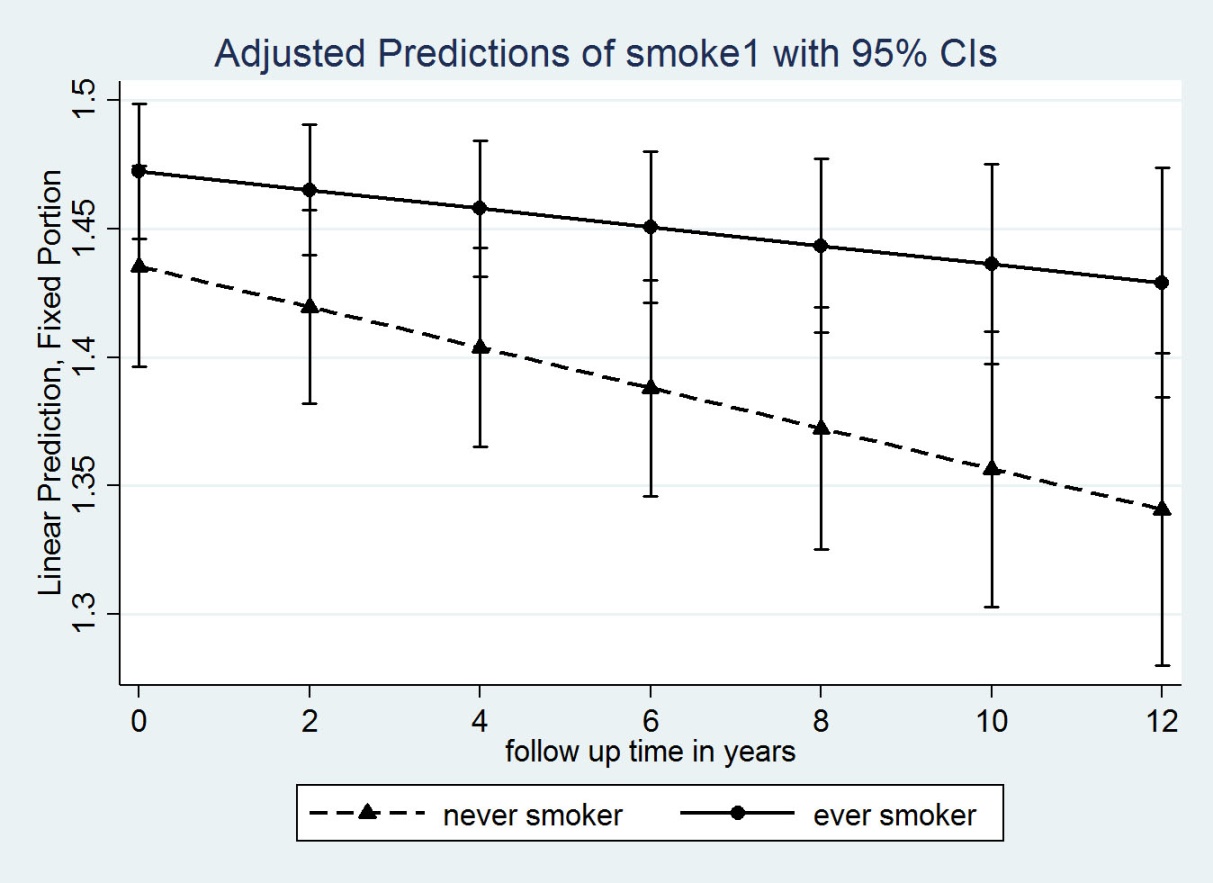

Supplement: S10 Fig — (DOCX) [file pone.0260229.s014.docx]

**Figure S11.**

NonHDL-C trajectories in men and women over the 12 years of follow up by smoking.


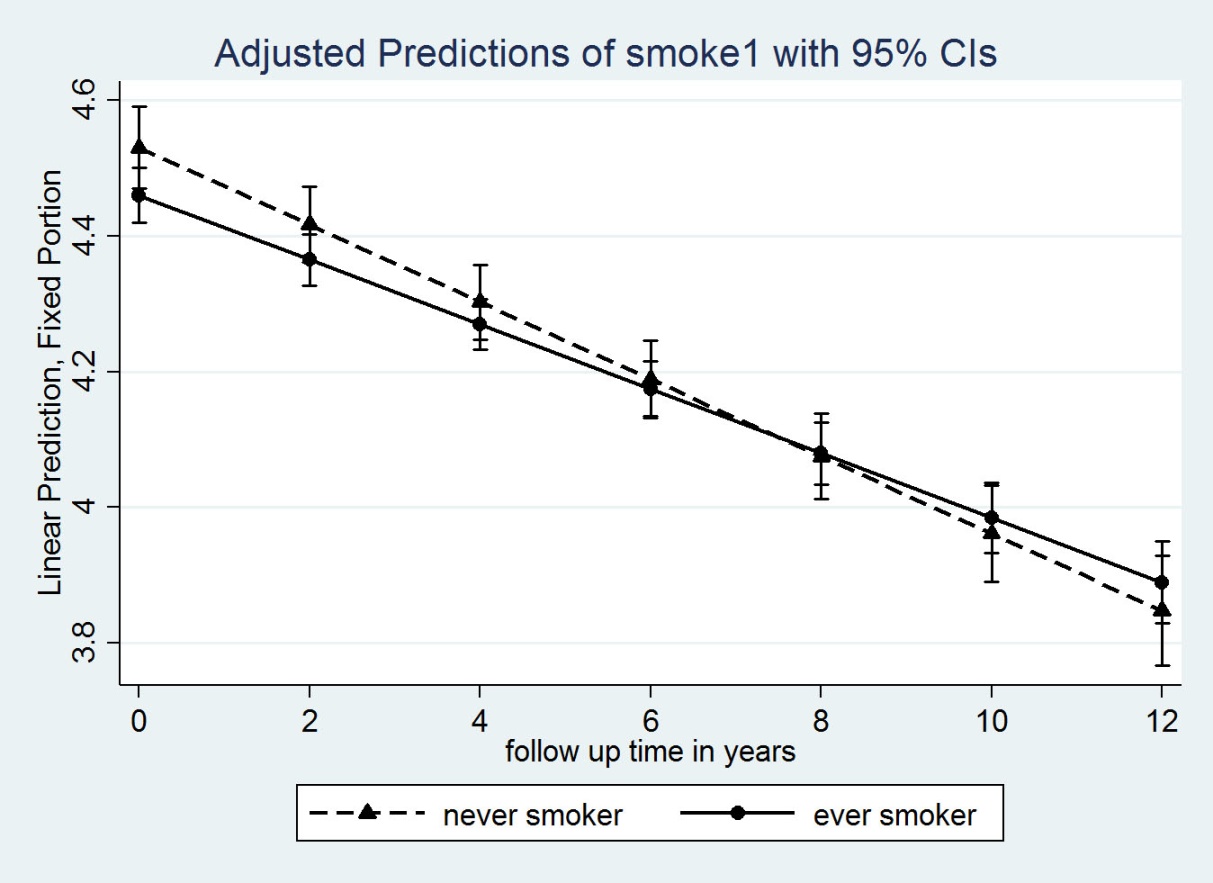

Supplement: S11 Fig — (DOCX) [file pone.0260229.s015.docx]

**Figure S12.**

Ratio LDL/HDL trajectories in men and women over the 12 years of follow up by smoking.


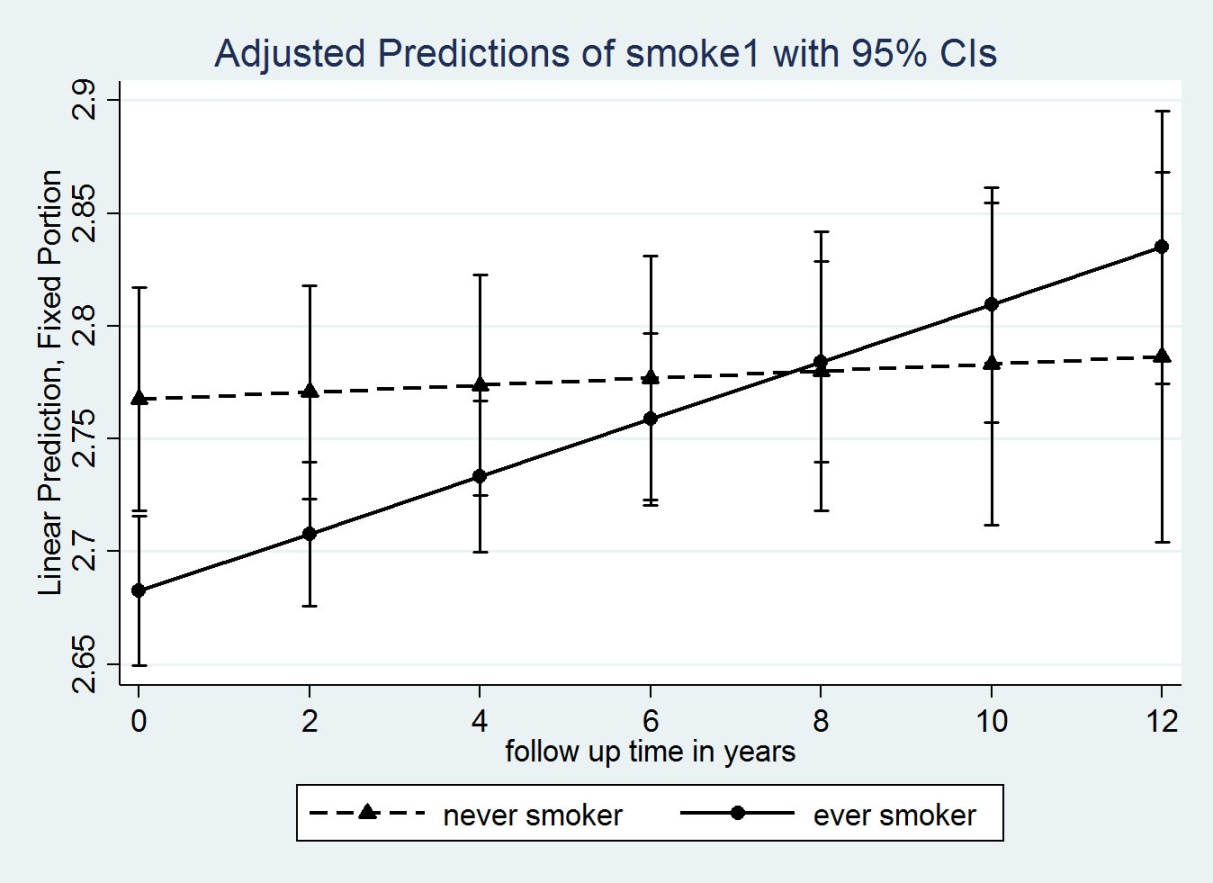

Supplement: S12 Fig — (DOCX) [file pone.0260229.s016.docx]
