# Supplementary material for: Longitudinal trajectories of blood lipid levels in an ageing population sample of Russian Western-Siberian urban population
Source: PLoS One. 2021 Dec 2;16(12):e0260229. doi: 10.1371/journal.pone.0260229 (PMC8638938; doi:10.1371/journal.pone.0260229)

**Figure S16.**

TG trajectories in men and women over the 12 years of follow up by lipid lowering treatment.


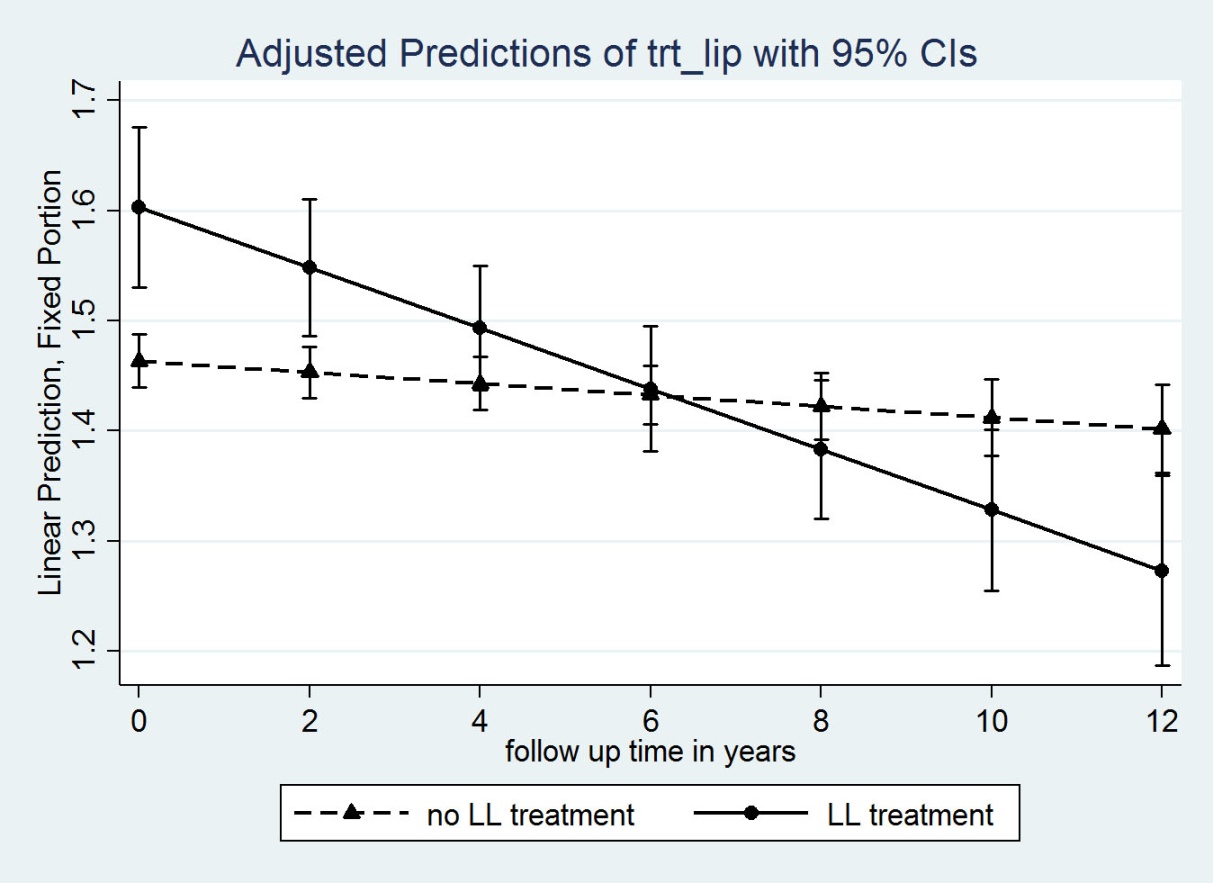

Supplement: S16 Fig — (DOCX) [file pone.0260229.s020.docx]
