# Supplementary material for: Longitudinal trajectories of blood lipid levels in an ageing population sample of Russian Western-Siberian urban population
Source: PLoS One. 2021 Dec 2;16(12):e0260229. doi: 10.1371/journal.pone.0260229 (PMC8638938; doi:10.1371/journal.pone.0260229)
Supplement: Attachment — Submitted filename: Response to Reviewers.docx [file pone.0260229.s023.docx]

**Comments to the Author**

1/ - it does not take into account a range of other confounding factors (diet particularities, other medications that may affect lipid blood levels, physical activity etc) that may affect the curve of the blood lipid changes

RE: We agree that in addition to lipid lowering treatment, age, sex and smoking, there are other factors that could influence plasma lipid levels in study participants. Concerning the lipid lowering treatment, as mentioned in the discussion, our subjects were almost exclusively treated by statins (no subjects on PCSK9 inhibitors or ezetimibe were included; see now added information at lines 460 - 463). In terms of other medications – unfortunately in the HAPIEE study we have no data available on specific medication of participants apart from lipid-lowering drugs, antihypertensive drugs or sugar lowering drugs (on dosage or regiment of intake). We have described this limitation of our data in the Discussion section of the revised manuscript.

In additional sensitivity analyses, we have further adjusted the models for fruit or vegetables intakes (by tertiles); total energy intake (centered at 10.6 MJ/day); and leisure time physical activity (less than 3 MET-hours/day; 3-10 MET hours/day; more than 10 MET hours/day). These adjustments did not materially change the results, including the direction of trajectories. We have added the description of additional adjustments to the revised paper (lines 353 - 366). Unfortunately, available data about dietary habits and/or general physical activities of examined subjects were not known in sufficient details at all three examinations to perform adequate analyses of effects. This is now listed between the study weaknesses (lines 414-416).

2/ - the mendelian randomization data about the life-time cardiovascular risk impact has to be taken into account

RE: Thank you for this comment raising the issue of Mendelian randomisation that we have now incorporated to the Discussion. We agree that Mendelian randomisation data could be of interest for the estimation of life exposure to the distinct risk factors and their life-time impact on CVD risk. However, such analysis has not been performed in our study, mainly because of unavailable genotyping data due to the shortage of funding, and this approach is out of the scope of present paper.

Nevertheless, it is merit to refer the recent papers which used the randomization by genetic score based on polymorphisms associated with LDL-C (known from GWAS), and reported the association between life-long LDL-C lowering and reduced CVD risk [Ference et al., 2019; Lind et al., 2021, ref list under 23 and 24]. In revised manuscript we have widened Discussion taking into account these findings (lines 384 - 389).

3/ - the results presented in the manuscript have to be supported by the data about the logic association of the blood lipid changes curve in different age groups with the prevalence of cardiovascular events in these groups.

RE: Thank you for this comment. In our analysis, we have taken into account CVD of participants by adjusting for CVD mortality rather than prevalence.

As mentioned in the Methods (lines – 161 - 162), Results (Table 4 and lines 242 - 247) and Discussion (lines – 337 - 345) sections, both total and cardiovascular mortality has been taken into account during the calculations – we have adjusted our results on these parameters. Two sensitivity analyses also suggest that the effect of CVD mortality on lipid trajectories is small and did not affect the observed trajectories substantially (see lines 340 - 345). Unadjusted and fully adjusted results are presented in Tables 2 and 4. In Tables 2, 3, 4 and wide list of supplementary figures, data are also presented for different age groups (as suggested by reviewer).
